# 

**Published:** 1861-06

**Authors:** 


### CONTENTS.

ORIGINAL ESSAYS AND COMMUNICATIONS.

Filling Teeth. By Dr. W. M. Rogers, .......... 325
Inflammation. By W. H. Atkinson .......... 338
Who are Dentists. By Wm. A. Pease .......... 365
Professional Advancement. By Dr. S. Clippinger .......... 369

PROCEEDINGS OF SOCIETIES.

Kentucky State Dental Association .......... 341

SELECTIONS.

Another "Lock-Jaw" Case .......... 371
Browne on Secretory Influences .......... 373
The Beard Question .......... 379
A New Anæsthetic .......... 380
The Surgical Diseases of the Tongue .......... 381
Editorial .......... 383

